# Needs and Needs Communication of Nursing Home Residents with Depressive Symptoms: A Qualitative Study

**DOI:** 10.3390/ijerph19063678

**Published:** 2022-03-19

**Authors:** Roxana Schweighart, Malte Klemmt, Silke Neuderth, Andrea Teti

**Affiliations:** 1 Institute of Gerontology, University of Vechta, 49377 Vechta, Germany; andrea.teti@uni-vechta.de; 2 Faculty of Applied Social Sciences, University of Applied Sciences Würzburg-Schweinfurt, 97070 Würzburg, Germany; malte.klemmt@fhws.de (M.K.); silke.neuderth@fhws.de (S.N.)

**Keywords:** depression, long-term care, person-centered care, needs assessment, needs fulfillment, healthy aging, well-being, quality of life, older adults

## Abstract

Nursing home residents are affected by depressive symptoms more often than elders living at home. There is a correlation between unmet needs and depression in nursing home residents, while met needs positively correlate with greater satisfaction and well-being. The study aims to examine the needs of nursing home residents with depressive symptoms and the communication of those needs, as no previous study has explicitly addressed the needs of this group of people and the way they are communicated. We conducted semi-structured interviews with 11 residents of three nursing homes and analyzed them using content-structuring content analysis. The residents reported diverse needs, assigned to 12 categories. In addition, barriers such as health impairments prevented the fulfillment of needs. As to the communication of needs, various interlocutors, facilitators, and barriers were identified. The findings reveal that residents can express their needs and are more likely to do so if the interlocutors are patient and take them seriously. However, lack of confidants, missing or non-functioning communication tools, impatience and perceived lack of understanding on the part of caregivers, and residents’ insecurities limit communication of needs.

## 1. Introduction

Worldwide, the proportion of older people in the population is rising [1]. Simultaneously, the number of people in need of care is also increasing. For example, about 800,000 people received inpatient care in Germany in 2019 [2]. Based on estimations, the number of required long-term care places will rise to 1.3 million by 2030 [3].

While the prevalence of depressive symptoms in the general population rises only marginally with an increase in age, the incidence of depressive disorders in nursing homes (NH) is relatively high. Between 10 to 30 percent of older people living at home show depressive symptoms, while it is nearly 50 percent among NH residents [4,5,6,7,8]. The essential risk factors for depression in NH residents are worsening cognitive impairment and decreasing functional ability [9]. Inadequate social contact also represents a central risk factor [10] for depression in nursing home residents. Studies have shown correlations between depression, aggressive behaviors, agitation, poor sleep quality, death wishes, and loneliness, among others, in NH residents [11,12,13,14,15]. Moreover, there is a relationship between depression and unmet needs [16,17]. Thus, it can be assumed that unmet needs are related to negative behaviors or conditions in nursing home residents. On the other hand, fulfilled needs positively impact depressive symptoms, well-being, and satisfaction in NH residents [18,19]. For needs to be met, it is relevant that residents communicate them. It is known that communication differs between older people with and without depression. As an example, one study [20] showed that verbal doctor–patient interaction is significantly shorter in older people with depression than in non-depressed older people. This allows the assumption that needs communication is also more limited in older people with depression than in older people without depression.

Only a few studies examined the needs of depressed older people. Stein and colleagues [17] identified the most unmet needs in the areas of physical health, mobility/falls, company, and psychological distress. However, no studies are explicitly known about the needs of depressed NH residents. In addition, there are no known studies examining how nursing home residents with depressive symptoms communicate their needs. Therefore, we chose a qualitative exploratory study design to examine the needs of NH residents with depressive symptoms and the communication. Improved needs communication may lead to better recognition and fulfillment of needs. In conclusion, understanding the needs as well as the needs communication of nursing home residents with depressive symptoms may lead to greater well-being and less depressiveness as well as increased person-centered care [21].

## 2. Materials and Methods

### 2.1. Study Design

In order to assess the subjective needs of NH residents and their expressions, an explorative cross-sectional study design was chosen by conducting 11 semi-structured interviews. The COREQ checklist was used to record and present the generated findings [22].

### 2.2. Sampling

The survey included residents from inpatient NH with a depressive disorder recorded in the patient’s file or judged to be depressed by caregivers. The professional judgement was based on their expertise and daily interactions and did not conduct screenings or validated assessments. Residents with insufficient communicative and cognitive abilities were excluded. Cognitive ability was also assessed by caregivers; no screening was performed. Respondents and their legal guardians were informed verbally and in writing about the project and asked for written consent. Recruitment of participants was difficult due to the SARS-CoV-2 pandemic, so the sample is limited to 11 participants. However, data analysis revealed repeated information, suggesting theoretical saturation.

### 2.3. Data Collection

The interviews were conducted from December 2020 to July 2021 in three NH in Würzburg, Southern Germany, under COVID-19 pandemic-related conditions such as wearing an FFP2 mask and keeping adequate distance. A negative PCR test for the interviewer was required at each interview to provide as much safety as possible for the residents. All interviews were conducted in the residents’ room or in a room made available for this purpose. The mean interview time was 41 min (min = 19 min, max = 74 min). An interview guideline, depicted in Table 1, was used. The guideline was developed by consensus of the authors and was pre-tested using the concurrent think-aloud approach.

This was followed by recording sociodemographic data and performing the Depression in Aging Scale (DIA-S) [23]. The DIA-S is an assessment tool that uses a ten-item questionnaire to provide information about depression or depressed mood that may be present in old age. A maximum of 10 points can be achieved. Zero to two points indicate inconspicuous mood. Three points or more suggest a suspicion of depression. At four points or more, depression of clinical significance is likely.

The first author is a social worker who works in a counseling center for people with mental health problems. All participants received the first author’s contact data in order to receive psychological support in case of stress due to the interview.

### 2.4. Data Analysis

The tape-recorded interviews were transcribed according to the rules of Dresing and Pehl [24]. Coding of the data material was performed deductively and inductively by two authors (R.S. and M.K.) independently, based on the content-structuring content analysis [25] with the procedure of consensual coding to provide intersubjective comprehensibility and transparency [26]. All data were coded with the final category system, using the MAXQDA 2020 software [27].

## 3. Results

The results include the sample description, followed by residents’ needs and barriers to need fulfillment in the first part. Subsequently, the needs communication as well as factors promoting and hindering communication are presented. The frequency of categories and subcategories can be found in the Supplementary Material Table S1.

### 3.1. Sample

Twelve residents were interviewed. The interview with one resident (B2) was terminated due to her health condition and was excluded from the analysis. The final sample consisted of 11 respondents (female: *n* = 5). The mean age of residents was 81.8 years at the time of the interviews (SD = 8.8; range = 71–93), and they lived in the NH, for a duration ranging from one month to almost six years. Six respondents had a depressive disorder documented in their patient file, while five residents were included based on the NH staff’s judgement. The mean severity of depressiveness was 5.9 on the DIA-S. The score of residents with a diagnosis (M = 6.0) is similar to the score of residents assessed as depressed by staff (M = 5.8). According to the DIA-S, all participants were very likely to be depressed (Score ≥ 4). For additional sample characteristics, see Table 2.

### 3.2. Perceived Needs and Barriers to Need Fulfillment

According to the residents’ statements, various needs were identified. Those were assigned to 12 categories and arranged alphabetically to avoid ranking the importance of the needs. Subsequently, barriers to need fulfillment could also be identified.

#### 3.2.1. Care Provision

The residents had needs as to nursing care, some partly fulfilled and some not fulfilled. Some residents noted that the nursing staff was kind and hardworking and paid attention to tidiness. Residents also reported feeling that staff were in a hurry, or were rude in their greetings and interactions. Moreover, they provided care too quickly and superficially, and did not respond to individual resident needs.

R5: “You know, they say they washed my hair today, the water was standing in the shower, and the toilet roll was completely soaked. I have now put it on the heating so that it dries again. And that goes just often once a little fast, and I say, there I’m not dry at all or what. That would be skin cream, that would not be wet. You get such answers then”.

The need for beauty and wellness was essential. Thus, the residents wanted to have their hair done and to use medical foot care. According to the residents, the services provided were sufficient to meet these needs.

#### 3.2.2. Dying and Death

A few residents addressed needs as to passing away and death, and the dying process, such as wanting to pass away quickly. In some cases, the need for dying was very clearly articulated.

R10: “I want to die. I want to close my eyes”.

One resident wanted to go to Switzerland to seek assisted suicide.

#### 3.2.3. Health

The need for improving one’s physical health condition was very important. Many respondents reported health problems, wishing that these problems were improved or even eliminated. Among these, orthopedic problems were repeatedly mentioned. The wish for more mobility went along with the wish for improved physical health. Moreover, residents also noted vision difficulties, bladder difficulties, gastrointestinal problems, dental problems, tumors, and the associated need for relief from these diseases or the pain that comes with it.

R4: “It doesn’t work as it did two years ago and so. I have such difficulties with my lower back. I had an operation on my spinal canal four years ago. And then I got three vertebrae stiffened, but I always try to walk early and go down to the garden. Because I don’t want to be in a wheelchair”.

Those residents reporting no serious physical health problems also wanted physical integrity and good health for the future.

I: “What do you wish for the future?”

R9: “That I stay reasonably healthy while I’m still alive”.

#### 3.2.4. Hobbies and Preferences

Respondents wanted to follow their hobbies and preferences. They stated that they wanted to watch movies, listen to music, read newspapers or books, go for walks and enjoy nature, play board games, drive a car, do crafts, cook, and bake, sing, discuss religious issues or politics, look out the window, and smoke cigarettes or drink alcohol. Some of these needs could be realized well in the NH and were, thus, met, such as listening to music or looking out of the window. Other needs, however, were deemed unrealistic by the residents. On the one hand, this was due to the individual characteristics of the residents, such as health limitations. On the other hand, structural and institutional factors were also mentioned as hindering the pursuit of individual hobbies. Examples included the lack of opportunity to cook or bake in the NH or the general prohibition on alcohol.

R1: “I’d like to have a little wine sometime. But we’re not allowed to drink alcohol in here, only non-alcoholic drinks”.

#### 3.2.5. Independence and Autonomy

The residents noted a loss of independence and autonomy after moving into the NH. They described that their independence was crucial to them before the transfer and that the loss of autonomy they experienced was stressful.

R10: “I just don’t want to be dependent on others; I don’t want that”.

One resident referred to the loss of autonomy and independence as why he could no longer enjoy his life. If residents could perform activities in the NH independently, they would like to do so. For example, some residents said they felt the need to wash themselves because they wanted to keep their independence, even if they required help with other tasks such as dressing and undressing.

#### 3.2.6. Move Out and Relocation

Some residents expressed the need to move out of the NH or to relocate within the home.

R5: “I really want to go back to my house, but my sons won’t allow it because they say you are alone there”.

The residents wished to live in their own homes again. Furthermore, they said that they could feel that living alone was no longer possible due to physical impairments, but concurrently they regretted it. Wishes to move in with relatives or to move to a NH closer to the living center of relatives were also stated. Three residents were living in double rooms at the time of the study. Two of these reported that they were unhappy with this situation, and they wished to move to a single room or to get another roommate.

#### 3.2.7. No Needs

Some residents stated no (more) needs. These respondents declared that any needs and wishes were meaningless because they could not be realized in the NH anyway, and they had to accept the situation.

R11: “Well, that doesn’t come true anyway. I can’t get out of here”.

However, when asked in more detail, all residents managed to name current needs.

#### 3.2.8. Occupation and Daily Structure

Several residents mentioned unmet needs for occupation offered on the part of the NH that would contribute to daily structure. As a result of these unmet needs, residents explained life to be monotonous and boring. The COVID-19 pandemic present at the time of the survey was repeatedly claimed as a reason. Due to the current restrictions, many occupational activities were either partially offered or not available at all.

R6: “Or the way we had it at the beginning, that’s all no longer allowed. That you had an occupation, everything no longer exists. I lie in my bed all day, then I watch TV, at night I watch TV”.

Another cited reason was that the NH staff were regularly ill or absent, meaning that scheduled activities were canceled.

#### 3.2.9. Psychological and Emotional Needs

The psychological and emotional needs were decisive for the respondents. Unfortunately, most of these needs were not fulfilled, according to the residents. Thus, they saw their lives as meaningless. Furthermore, they felt alone and lonely, and they were afraid, sad, dissatisfied, and frustrated. In addition, they had the feeling of having to sacrifice their personality, that self-doubt and anger plagued them, and that their lives did not live up to their expectations.

R7: “Life is frustrating as hell. From top to bottom. Because I’m locked in here, feeling locked in, and I’m somewhat losing consistency right now”.

However, 2 of 11 residents interviewed stated that they felt generally comfortable and satisfied in the NH.

#### 3.2.10. Reminiscence

One need that was not specifically mentioned by residents, but was coded, was the need for reminiscence and a review of past life. Some of the residents did not respond to the questions but shared their memories and experiences. Nine respondents recapitulated anecdotes from their lives before moving into the NH, many of which related to periods relevant to the residents, including their school years, youth, or marriage. These parts of the conversation were interpreted as residents wishing to reflect on their lives and to pass on what they had experienced.

I: “And if you now recall a situation, where you talked about your needs, what did that look like? Can you tell me about it?”.

R1: “It’s like this, in my youth, I didn’t have anything. I was born, I didn’t know my father. He died, and my mother raised me, then I started school at the age of seven. The first class was bad, right away, I had to do it twice...”.

#### 3.2.11. Service and Facilities

Several of the needs were related to the service situation in the NH, such as catering, service offerings, and the NH’s facilities. These needs were considered as partially met or not met. However, residents sometimes declared that the food tasted good, and they liked the orderliness and the services offered in the NH, such as the laundry service.

R12: “I want to stay here, I have my food there. And I get my laundry ironed and washed”.

Other residents reported unmet needs, specifically regarding culinary supply. They explained that the food was not to their liking or there was either too much or too little of it, causing one resident to report her being hungry at night.

#### 3.2.12. Social Needs

Social needs were mentioned most frequently and were addressed by all residents. The residents often described a desire for more social contact, showing that the need was, thus, not completely met. The wishes for more contact were directed at family, relatives, friends, acquaintances, other residents, and also at people from the formal circle of professionals, including a legal guardian, a psychologist, or a social worker from a counseling center. Residents reported mental stress due to a lack of social contact.

R7: “I sit here alone all afternoon. And then I get the big cry here (crying)”.

Concurrently, they emphasized the value of the existing contact with confidants and the exchange with other people and that this was crucial for a satisfied life in the NH. However, as it came to other residents, some respondents found the existing social contacts to some extent as burdensome. The residents living in a double room were dissatisfied due to problems and disagreements with the roommate.

R10: “But it’s quite bad. And I have nothing against the man lying with me, but I can hardly sleep at night. He always gets coughing attacks, and it’s loud, loud with him. Well, maybe I also have something that others don’t like. And he always wants to play, always wants to play”.

Furthermore, several interviewees stated that they could not identify with other residents and found them exhausting and did not want to have much contact with them.

#### 3.2.13. Barriers to Need Fulfillment

The residents quoted various explanations why some needs were not met. Among other things, the NH staff were a barrier, according to the interviewees. For example, one resident stated that NH staff did not take her psychological-emotional needs seriously, which resulted in her current need for safety not being met.

R5: “Well, I’m often very dizzy and therefore very anxious. And they sometimes don’t take that too seriously”.

However, residents’ limitations and health problems also explained why specific needs could no longer be met. Among these limitations, residents listed limited mobility, diminished concentration ability, pain, gastrointestinal problems, and eye diseases. Apart from these two barriers, the residents also mentioned factors related to the NH, such as the absence of single rooms or rules that exist in the NH. They also noted relatives who made decisions against the residents’ will and an absence of social contacts or negative social experiences, all of which could be a barrier. Ultimately, the residents also mentioned the COVID-19 pandemic and the associated limitations, blocking the residents’ needs.

### 3.3. Communication of Needs

Most residents stated that they mentioned their needs to other people. One resident, instead, said that she had nobody to talk about her needs and another resident pointed out that he did not express his needs because it was pointless. In addition, he was afraid to argue with his family as he moved to the NH against his will.

R8: “For certain reasons, this time, and for the first time in my life, I must say, I kept silent in order not to let events occur that I could see coming. So it probably wouldn’t have gone very well with the family”.

#### 3.3.1. Interlocutors

The residents disclosing their needs named several interlocutors. Other residents, acquaintances, relatives, family, and the NH staff, including the nursing staff, were referred to as interlocutors in the communication of needs. However, residents also directed their needs to doctors, legal guardians, psychologists, and social workers. One resident explained a situation where he also told strangers about his need to die.

R10: “The ones who drove me in here were two such young girls, I thought they were still schoolgirls. So I said, you don’t have to drive me to Würzburg, just take me straight to the cemetery”.

#### 3.3.2. Enablers to Communication

Some facilitating factors could be identified. For instance, one resident noted that he was more successful in communicating his needs because he felt he had arrived at the NH and had made social contacts. Other residents also indicated that good social connections were supportive in communicating their needs, as were staff who were trusting, friendly, and patient.

R1: “They are very nice, you can talk to them. They also listen and do things for us when we need something. And then they say, that works, I’ll get that for you. And that’s where I get it done”.

#### 3.3.3. Barriers to Communication

In addition to the facilitating factors, residents reported barriers to the communication of needs. Some residents said that they had no confidants with whom they could speak in detail about their concerns. When communication tools were unavailable or not functioning, smooth communication was also not possible.

R6: “And I can’t make phone calls because I don’t have the money, it’s too expensive. And what am I supposed to do? Letters are no longer written. I don’t have a computer either, that you could computerize. I don’t understand anything about it either”.

As to the nursing staff, the residents remarked that they often had no time or even no desire to communicate. According to the residents, the staff were overworked and reacted to expressed needs with incomprehension and irritation. As a consequence, residents kept their needs to themselves.

R3: “They don’t have time. You saw that, she comes in, and she’s out again. You can’t gasp that fast”.

Insecurities of the residents played a critical role, causing them to keep quiet rather than speak openly about their needs. Therefore, the respondents remarked that they did not dare to address specific needs because they were afraid that they could say something wrong. In addition, residents felt that not everything could be said in NH due to concerns that the content of the conversation would not be confidential.

R12: “When I talk to one, I know exactly whom I’m talking to; I don’t do that with everyone. If I know they’re going to keep it to themselves, so you can’t say it to everybody, it doesn’t fit”.

The feeling of being a burden or a nuisance for the other person also caused residents to keep their needs to themselves. Moreover, respondents doubted their needs and minimized them. For example, one resident uttered that she was ashamed of herself because all she did was complain, and the other thought that she was too demanding. In the cases of both residents, these attitudes made them regard their needs as unjustified and not expressed, or they expressed them only reluctantly. Lastly, the feeling that expressing their needs would not lead to any change also caused residents to avoid communicating them.

## 4. Discussion

This study aims to examine the needs of NH residents with depressive symptoms and the communication of these needs. Two major themes were identified. The first theme includes needs and barriers to need fulfillment. The second theme is about needs communication, including interlocutors, facilitators, and barriers.

Within the first theme, we found diverse and complex needs of the residents, assigned to 12 categories. Specifically, the residents’ psychological-emotional and social needs played a critical role. As to those, residents essentially described unmet needs. According to the literature, inadequate social contact is a central risk factor for depression in NH residents [10]. In addition, unmet emotional and psychological needs can be expected to impact existing depressive symptoms negatively.

Furthermore, barriers to the fulfillment of needs were identified. On the one hand, these included external ones, such as behaviors and statements of caregivers and decisions of relatives. On the other hand, internal factors, such as poor health, were mentioned. Even though some of these factors, including severe physical limitations, are very difficult to change, removing some barriers might ease need fulfillment. Training programs for caregivers, for instance, can stimulate their positive attitudes toward residents, causing staff to take residents’ needs more seriously and to address them adequately [28]. Questioning and eventually changing some of the existing rules in NH can also contribute to more self-determination and fulfillment of needs. Especially during the COVID-19 pandemic, regulations in German NH were sometimes very strict, leading to distress among nursing home residents with depressive symptoms [29].

Within the second theme, interlocutors could first be identified. If there was communication of needs, it was toward diverse groups of people. The results show that the residents opened up to close confidants and the staff, or even to strangers. In most interview situations, the interviewees communicated very openly and were glad that someone spoke with them about their needs. In contrast, some residents expressed at the outset that they no longer had any needs, owing to feelings of futility and resignation. However, with patience and interest, even those residents began expressing themselves. These results reveal that depressive NH residents are conscious of their needs and can disclose them. Shiells and colleagues’ scoping review [30] showed that not only can residents without cognitive impairment articulate their needs, but that residents with dementia have this ability as well.

It was beneficial for the residents to communicate their needs when there existed a trusting relationship with the caregivers. Studies show that effective communication between residents and caregivers is positively associated with a higher quality of life, lower depression, and less verbal and physical aggression [31,32]. Communication training involving person-centered interventions can also help in this regard. Examples of effective interventions that can improve the relationship between caregivers and residents include regular one-to-one contact between residents and caregivers, as well as implementing biographical approaches such as storytelling [33,34]. Improved relationship and communication between caregivers and residents can lead to more needs communication and fewer unmet needs for residents [35,36]. For those who feel that their needs are not heard and that addressing these would not cause a change, communicating them is futile [37]. However, the malfunctioning of communication tools, the absence of confidants, the sentiment that staff had neither the time nor the inclination to talk, and the insecurities of the residents were barriers to the expression of needs. Under this, installing functioning communication tools, such as tablets or free-of-charge telephones, could be implemented quite easily in NH. Residents’ depressive symptoms can explain the existing insecurities. For instance, depressive illness causes decreased self-esteem and self-confidence, social withdrawal, and resignation [38]. Due to this sense of worthlessness, needs can be acknowledged as irrelevant and, thus, not communicated. Likewise, the social withdrawal and resignation accompanying depression can make needs communication especially difficult for residents with depressive symptoms. Psychotherapy and/or drug therapy can, thus, be helpful in reducing depressive symptoms, making it easier for residents to communicate their needs [39,40]. Only expressed needs can be identified and addressed. When needs are fulfilled, this positively affects depressive symptoms, well-being, and satisfaction [18,19]. However, the unmet needs of NH residents are associated with existing depression [41].

NH are known to have a high workload, and there is often little time available to have longer-lasting conversations with the residents. The use of assessment tools, such as the Preferences for Everyday Living Inventory–Nursing Home (PELI–NH) questionnaire [42] or the Camberwell Assessment of Need for the Elderly (CANE) questionnaire [43], can help conduct a needs assessment. Such instruments can be beneficial to detect current needs and provide an overview. Nevertheless, in-depth and recurrent conversations with residents are crucial to address the high complexity and individuality of needs so that results can be validated and unmet needs uncovered.

Our study has some limitations. First, the concept of needs is very complicated and there exists no agreed definition [44]. A common definition was established in advance to ensure that all participants understand the same by the concept of needs. Moreover, the NH staff performed the selection of respondents due to the COVID-19 pandemic, as access to the NH was restricted, and the risk to residents had to be kept to a minimum. The pandemic may also have caused some bias in data. Residents may have described prevalent needs because of the pandemic that would not be relevant without it. Visitation restrictions and the absence of activities may have resulted in excessive unmet social needs and needs for occupation. Additionally, residents and the interviewer wearing a face mask may have inflicted further limitations in the interview situation because some facial expressions were not evident. As for the sample, it must also be said that it is relatively small. The objective of the present study was to illuminate and reconstruct the subjective views of nursing home residents with depressive symptoms. In contrast to quantitative research aiming to obtain statistically representative results, our qualitative study did not aim to be representative. Furthermore, it must be stated that the DIA-S is a short screening, not a comprehensive depression diagnosis. The DIA-S cannot substitute for a medical diagnosis or a structured clinical interview and only indicates the presence of a depressive disorder based on the presence of depressive symptoms. Due to the qualitative cross-sectional design, no statements can be made about whether the unmet needs caused the depressive symptoms or whether the unmet needs were present because of the depressive symptoms. Finally, no residents without depressive symptoms participated in the study. The question arises whether residents without depressive symptoms experience other needs and communicate them differently. No statement is available on this.

## 5. Conclusions

This study shows that NH residents with depressive symptoms have diverse and complex needs, many of which are unmet. They can communicate these needs and may want to do so if their interlocutor is patient, takes them seriously, and is interested in what they are about to say. Especially for residents with depressive symptoms, communicating needs can be more challenging than for NH residents without depressive disorder due to social withdrawal, resignation, and low self-esteem, which can cause an increase in unmet needs. The unmet needs require serious attention because unfulfilled needs and depressive symptoms can negatively affect each other, resulting in a downward spiral. The depressed residents should receive individual, sensitive, and patient attention to help them communicate their needs and preferences. Conversations with residents should repeat at regular intervals to identify and address changes in needs. Barriers to needs communication and fulfillment should be defined and progressively removed if possible. The NH is the last stage of life for many residents before death. There, they should enjoy a fulfilled and contented life. The fulfillment of individual demands and needs is crucial for this.

## Figures and Tables

**Table 1:** Interview guideline.

(1) What has your life been like since moving into the NH?	
(2) What is important to you for your well-being and satisfaction?	
(3) What needs do you feel in your current life?	
(4) Do you talk about your needs with other people?	
If question (4) is answered “No”	If question (4) is answered “Yes”
(5a) What are some reasons that you do not talk about your needs?	(5b) With whom do you talk about your needs?
(6a) Which people would you be most likely to talk to about your needs?	(6b) What needs do you find easier/harder to talk about?
(7a) What would help you feel more comfortable talking about your needs?	(7b) What makes it easier/more difficult for you to talk about your needs?
(8) What do you wish for the future?	

**Table 2:** Sample Description.

Abbreviation	Sex ^a^	Age ^b^	Documented Diagnosis ^c^	Marital Status, Children	Length of Stay ^d^	Score DIA-S ^e^
R1	M	71	Major depressive episode with psychotic symptoms	Widowed, 0	53	4
R3	F	75	Depression	Widowed, 1	9	8
R4	F	83	Depression	Widowed, 2	23	4
R5	F	90	Major depressive episode	Widowed, 2	2	4
R6	F	73	Depression	Divorced, 3	30	9
R7	M	76	n.a. ^f^	Widowed, 0	6	5
R8	M	93	n.a.	Widowed, 1	36	6
R9	F	90	n.a.	Widowed, 0	9	5
R10	M	86	n.a.	Single, 0	1	8
R11	M	92	n.a.	Married, 1	36	5
R12	M	71	Major depressive episode	Divorced, 3	70	7

^a^ F = female; M = male; ^b^ in years; ^c^ as noted in patient file; ^d^ From moving in to the interview in months; ^e^ 0–2 points: inconspicuous mood; 3 points or more: suspicion of depression; 4 points or more: depression of clinical significance is probable; ^f^ n.a., not available.

## Data Availability

The data that support the findings of this study are available from the corresponding author upon request.

## Supplementary Materials

The following supporting information can be downloaded at: https://www.mdpi.com/article/10.3390/ijerph19063678/s1, Table S1: Frequencies of categories and subcategories by residents.
