# Supplementary material for: Needs and Needs Communication of Nursing Home Residents with Depressive Symptoms: A Qualitative Study
Source: Int J Environ Res Public Health. 2022 Mar 19;19(6):3678. doi: 10.3390/ijerph19063678 (PMC8949340; doi:10.3390/ijerph19063678)
Supplement: Supplementary file 1 [file ijerph-19-03678-s001.zip › ijerph-1611929-supplementary.pdf]

Supplement Table S1. Frequencies of Categories and Subcategories by Residents.

**Main Theme**	**Main Category**	**Subcategory**	**Number of Codings**	**Residents (n = 11)**
**Needs and Need Fulfillment**	Perceived Needs	Care Provision	22	6
		Dying and Death	8	4
		Health	33	11
		Hobbies and Preferences	40	10
		Independence and Autonomy	21	10
		Move out and Relocation	15	6
		No Needs	17	3
		Occupation and Daily Structure	18	9
		Psychological and Emotional Needs	65	10
		Reminiscence	47	9
		Service and Facilities	33	9
		Social Needs	80	11
	Barriers to Need Fulfillment	Covid Pandemic	19	8
		Health and Interpersonal Barriers	35	8
		NH Staff	20	6
		Regulations and Circumstances within the NH	25	9

		Relatives and Family	15	3
		Social Contacts	6	2
**Needs Communication**	Interlocutors	Acquaintances	5	3
		NH Staff	13	7
		Other Residents	1	1
		Professional Helpers	5	3
		Relatives and Family	9	6
		Strangers	1	1
	Facilitators to Communication	NH Staff	10	7
		Sense of Having Arrived in the NH	1	1
		Social Contacts	13	8
	Barriers to Communication	Insecurities and Interpersonal Barriers	14	5
		Lack of Interlocutors	4	3
		Missing/Non-functioning Communication Tools	3	2
		NH Staff	20	7
		Pointlessness	12	3
